# Patient-Specific Exercises with the Development of an End-Effector Type Upper Limb Rehabilitation Robot

**DOI:** 10.1155/2022/4125606

**Published:** 2022-10-27

**Authors:** Mingjie Dong, Wenpei Fan, Jianfeng Li, Pengfei Zhang

**Affiliations:** Faculty of Materials and Manufacturing, Beijing University of Technology, No. 100 Pingleiyuan Chaoyang District, Beijing 100124, China

## Abstract

End-effector type upper limb rehabilitation robots (ULRRs) are connected to patients at one distal point, making them have simple structures and less complex control algorithms, and they can avoid abnormal motion and posture of the target anatomical joints and specific muscles. Given that the end-effector type ULRR focuses more on the rehabilitation of the combined motion of upper limb chain, assisting the patient to perform collaborative tasks, and its intervention has some advantages than the exoskeleton type ULRR, we developed a novel three-degree-of-freedom (DOF) end-effector type ULRR. The advantage of the mechanical design is that the designed end-effector type ULRR can achieve three DOFs by using a four-bar mechanism and a lifting mechanism; we also developed the patient-specific exercises including patient-passive exercise and patient-cooperative exercise, and the advantage of the developed patient-cooperative exercise is that we simplified the human-robot coupling system model into a single spring system instead of the mass-spring-damp system, which efficiently improved the response speed of the control system. In terms of the organization structure of the work, we introduced the end-effector type ULRR's mechanical design, control system, inverse solution of positions, patient-passive exercise based on the inverse solution of positions and the linear position interpolation of servo drives, and patient-cooperative exercise based on the spring model, in sequence. Experiments with three healthy subjects have been conducted, with results showing good trajectory tracking performance in patient-passive exercise and showing effective, flexible, and good real-time interactive performance in patient-cooperative exercise.

## 1. Introduction

There are more and more people in the world suffering from spinal cord injuries (SCIs), with approximately 60% with cervical SCIs leading to tetraplegia [1], which can create severe arm disabilities, resulting in an inability to complete activities of daily living (ADLs) [2]. In addition, stroke is becoming the leading cause of permanent disabilities worldwide, with over 15 million new cases each year and 50 million stroke survivors [3]; more than two-third of all patients affected by stroke have impaired upper limb motor function and have difficulty in independently performing ADLs [4]. Evidence has suggested that upper limb motor skills can be improved by following rehabilitation interventions [5], which attracts more and more scholars engaged in upper limb rehabilitation robot (ULRR) research [6, 7], for the ULRRs have the potential to provide intensive rehabilitation consistently for a longer duration [8] irrespective of the skills and fatigue level of the therapist.

There are two types of ULRRs that have been studied the most, the exoskeleton type and end-effector type [9]. For the exoskeleton-based ULRR, the robots can resemble human limbs as they are connected to patients at multiple points and their joint axes match with human joint axes; training of specific muscles by controlling joint movements at calculated torques is possible, and the number of anatomical movements can exceed six; typical exoskeleton ULRRs are SUEFUL-7 [10], CADEN-7 [11], ARMin III [12], L-EXOS [13], ExoRob [14], RUPERT [15, 16], BONES [17], ULEL [18], and Limpact [19]; nonetheless, increasing the number of movement parts increases the number of device modules, so the system setup becomes difficult; moreover, since the shoulder has a variable joint center, the mechanical design and control algorithms become more complicated [7]. By comparison, the end-effector type ULRRs are connected to patients at one distal point, and their joints do not match with human joints; force generated at the distal interface changes the positions of other joints simultaneously, making isolated movement of a single joint difficult [20, 21]; the advantages of the end-effector type ULRRs are that they have a simple structure and less complex control algorithms and can avoid abnormal motion and posture of the target anatomical joints and specific muscles; typical end-effector type ULRRs are MIT-Manus [22], AMES [23], iPAM [24], PASCAL [25], Fourier M2 [26], EEULRebot [27], hCAAR [28], PARM [29], CASIA-ARM [30], Sophia-3 [31], and BULReD [2, 32].

Recent study found that the end-effector type ULRR intervention is better than the exoskeleton type ULRR intervention with regard to activity and participation among chronic stroke patients with moderate-to-severe impairment of upper extremity function after four weeks of intervention [9]. In addition, research studies have shown that the shoulder complex of upper limb is very complex, and it is difficult to design an exoskeleton type ULRR that is compatible with the upper limb of users because the glenohumeral joint moves with the functional relevance of the shoulder girdle during humeral elevation and the glenohumeral joint has three degrees of freedom (DOFs) with axes intersecting perpendicularly in the glenohumeral joint center, which is obviously a spherical joint and can be regarded with a generalized glenohumeral joint with floating center [7, 33]. Also, the end-effector type ULRRs focus more on the rehabilitation of the combined motion of upper limb chain and assist the patient to perform collaborative tasks; they are safer compared with exoskeletons because they are not in direct contact with the human body.

In view of the abovementioned advantages, we developed a three-DOF end-effector type ULRR based on the optimum design of a five-bar linkage mechanism [34] and aimed to realize the patient-specific exercises based on our previous work on interactive control of rehabilitation robots [35, 36]. Our end-effector type ULRR is developed for neurorehabilitation to help patients regain motor function following a neurological condition or injury for wide range of motor impairments including stroke, cerebral palsy, SCI, multiple sclerosis, Parkinson's disease, hemiplegic shoulder pain, and muscle spasticity. The remainder of the paper is organized as follows. The three-DOF end-effector type ULRR with its mechanical design, control system, and inverse solution of positions is introduced in Section 2. The patient-specific exercises including patient-passive exercise and patient-cooperative exercise are described in Section 3.  Section 4 demonstrates the experiments and results of the patient-specific exercises with the developed end-effector type ULRR, while conclusions and future works are summarized in Section 5.

## 2. Three-DOF End-Effector-Based ULRR

### 2.1. Mechanical Design and Control System

**Figure 1:**
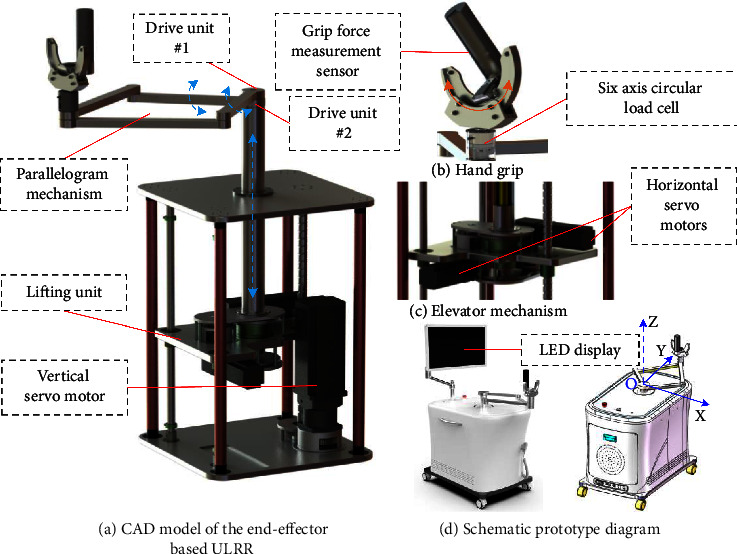
Mechanism and prototype of the developed end-effector type ULRR.

The computer-aided design (CAD) of the developed end-effector type ULRR is shown in Figure 1, which has three DOFs, with two DOFs in the horizontal plane and one DOF in the vertical direction. The advantage of the mechanical design is that the designed end-effector type ULRR can achieve three DOFs by using a four-bar mechanism and a lifting mechanism. The horizontal motion is achieved with the two drive units of the parallelogram mechanism driven by two DC servo motors (SDGA-04C11AB, TODE, China) and two right-angle reducers (PXW60, Times Brilliant, China) with a reduction ratio of 30 : 1; and the rotation axes of the two drive units are concentric to make the mechanism compact and simple. The vertical motion is driven by an AC servo motor (SMH110 1573028EBM-1, Kinavo, China) and a planetary reducer (KPLF090, Kofon, China) with a reduction ratio of 3 : 1. The three servo motors can ensure that the ULRR moves in three dimensions, with three incremental encoders (2500 pulse/revolution) as feedback. The handle grip is designed with two passive joints to make the wrist part flexible and comfortable, and a grip force measurement sensor (FFK–300N, Forsentek, China, with nonlinearity ±0.3% of rated output, hysteresis ±0.3% of rated output, and nonrepeatability ±0.2% of rated output) is deployed to measure the grip force in real time. In addition, a six-axis circular load cell (M3703C, SRI, China, with capacity of 200N, crosstalk of 2%F.S., nonlinearity of 0.5%F.S., and hysteresis of 0.5%F.S., where F.S. represents the full scale) is set up between the handle grip and the parallelogram mechanism to detect the interaction force and torque by using a data acquisition card (M8128B1, SRI, China, 24 bit sigma-delta ADC, sampling rate up to 2kHz, and resolution of 1/5000 to 1/10000 of full scale). For safety, mechanical limits are installed on the transmission gears to constrain the workspace of the three DOFs, in which the screw bolts serve as swing pins to facilitate mechanical limit switching.

**Figure 2:**
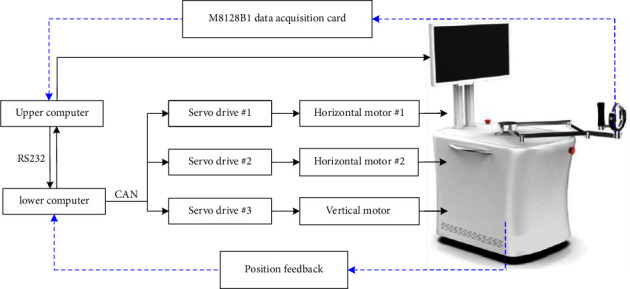
Schematic diagram of the control system.

The two DC servo motors are driven by two DGFxo DX060 drives (HDT, Italy), which have good interpolated position modes using the CANopen DS402 Protocol. Also, the AC servo motor is driven by one CDHD-006 servo drive (Servotronix Motion Control Ltd., Israel), which also has interpolated position mode using CANopen Protocol. Besides, there is also a LED display used to display the human-machine interface (HMI), carry out patient-active rehabilitation training, promote human-robot interaction, and improve the effect of rehabilitation training. The schematic diagram of the control system is shown in Figure 2; the upper computer and the lower computer communicate through RS232, and the lower computer and the driver communicate through CAN bus; all the control algorithms are completed on the upper computer coded by using C#. Also, we know that accurate trajectory tracking control is necessary in both patient-passive and patient-cooperative exercises. The inverse solution of the positions of the end-effector type ULRR must be solved first, in order to achieve high-precision trajectory tracking control.

### 2.2. Inverse Solution of Positions

From Figure 1, we can see that the motion in vertical direction is only driven by an AC servo motor, while the horizontal motion is accomplished through the resultant motion of two rotational axes, drive unit #1 and drive unit #2. Therefore, there is no coupling relationship between the movement in the vertical direction and the movement on the horizontal plane, and we mainly analyze the horizontal two-dimensional motion for the inverse solution of positions; the three-dimensional motion can be solved by the combination of horizontal two-dimensional motion and vertical motion.

**Figure 3:**
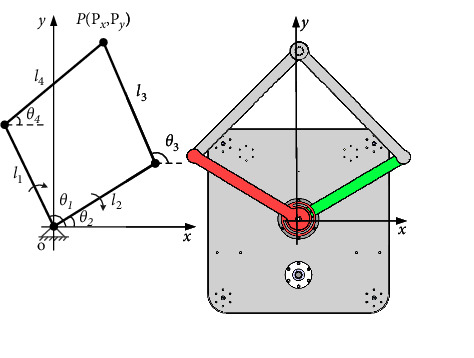
Kinematics model in horizontal direction.

The kinematics model of the developed end-effector based ULRR in horizontal direction is shown in Figure 3, where *l*_1_ and *l*_2_ are the rod lengths of the corresponding drive unit #1 and drive unit #2, respectively; *θ*_1_, *θ*_2_, *θ*_3_, and *θ*_4_ are the angles between each link and the *X*-axis, and the definition of the coordinate system is shown in Figure 1(d).

The lengths of the four connecting rods of the parallelogram mechanism are designed as in (1), and the position inverse solution is to solve the rotation angle of the two driving units *θ*_1_ and *θ*_2_, through the known position of point *P*.

(1) $$\begin{array}{c} \left\{\begin{array}{c} l_{1}=l_{2}=315mm,\\ l_{3}=l_{4}=385mm. \end{array}\right) \end{array}$$

Firstly, we can deduce the relationship between *P* and *θ*_1_. According to Figure 3, we can obtain the relationship equation of point *P*(*P*_*x*_, *P*_*y*_) and *l*_1_, *l*_4_, *θ*_1_, *θ*_4_, as shown in the following equation:

(2) $$\begin{array}{c} \left\{\begin{array}{c} P_{x}=l_{1}\cos\theta_{1}+l_{4}\cos\theta_{4},\\ P_{y}=l_{1}\sin\theta_{1}+l_{4}\sin\theta_{4}. \end{array}\right) \end{array}$$

(2) can be transformed into the following:

(3) $$\begin{array}{c} P_{x}^{2}+P_{y}^{2}+l_{1}^{2}-l_{4}^{2}-2P_{x}l_{1}\cos\theta_{1}-2P_{y}l_{1}\sin\theta_{1}=0\text{.} \end{array}$$

Let us define *x*_1_=tan*θ*_1_/2; then, we can get

(4) $$\begin{array}{c} \left\{\begin{array}{c} \sin\theta_{1}=\frac{2x_{1}}{1+x_{1}^{2}}\\ \cos\theta_{1}=\frac{1-x_{1}^{2}}{1+x_{1}^{2}} \end{array}\right),\;\left(\theta_{1}\neq 2k\pi +\pi ,k\in Z\right)\text{.} \end{array}$$

Putting (4) into (3), we can get

(5) $$\begin{array}{c} P_{x}^{2}+P_{y}^{2}+l_{1}^{2}-l_{4}^{2}-2P_{x}l_{1}\frac{1-x_{1}^{2}}{1+x_{1}^{2}}-2P_{y}l_{1}\frac{2x_{1}}{1+x_{1}^{2}}=0\text{.} \end{array}$$

Defining *A*_1_=*P*_*x*_^2^+*P*_*y*_^2^+*l*_1_^2^ − *l*_4_^2^, *B*_1_=−2*P*_*x*_*l*_1_, and *C*_1_=−2*P*_*y*_*l*_1_, we can obtain

(6) $$\begin{array}{c} A_{1}+B_{1}\frac{1-x_{1}^{2}}{1+x_{1}^{2}}+C_{1}\frac{2x_{1}}{1+x_{1}^{2}}=0\text{.} \end{array}$$

We can deduce (7) when *B*_1_^2^+*C*_1_^2^ − *A*_1_^2^ > 0.

(7) $$\begin{array}{c} \left\{\begin{array}{c} x_{1}=\frac{-C_{1}\pm \sqrt{B_{1}^{2}+C_{1}^{2}-A_{1}^{2}}}{A_{1}-B_{1}}\text{,}\\ \theta_{1}=\frac{180}{\pi }*2\;\arctan{\;x}_{1}\text{.} \end{array}\right) \end{array}$$

Similarly, we can obtain the relationship equation of point *P*(*P*_*x*_, *P*_*y*_) and *l*_2_, *l*_3_, *θ*_2_, *θ*_3_, as follows:

(8) $$\begin{array}{c} \left\{\begin{array}{c} P_{x}=l_{2}\cos\theta_{2}+l_{3}\cos\theta_{3}\text{,}\\ P_{y}=l_{2}\sin\theta_{2}+l_{3}\sin\theta_{3}\text{.} \end{array}\right) \end{array}$$

We can also deduce (9) when *B*_2_^2^+*C*_2_^2^ − *A*_2_^2^ > 0.

(9) $$\begin{array}{c} \left\{\begin{array}{c} x_{2}=\frac{-C_{1}\pm \sqrt{B_{1}^{2}+C_{1}^{2}-A_{1}^{2}}}{A_{1}-B_{1}}\text{,}\\ \theta_{2}=\frac{180}{\pi }*2\;\arctan{\;x}_{1}\text{.} \end{array}\right) \end{array}$$

Since there is a symmetrical rod length relationship in the four-bar linkage parallelogram mechanism, the solutions of (7) and (9) are the inverse solutions of positions. For the reason that *θ*_1_ is always bigger than *θ*_2_, the inverse solution of positions of the horizontal two-dimensional motion of the developed end-effector type ULRR is as in (10) and (11), where *θ*_1−value_ and *θ*_2−value_ are the inverse solutions of positions of *θ*_1_ and *θ*_2_, respectively.

(10) $$\begin{array}{c} \theta_{1-value}=\left\{\begin{array}{c} \max\;\left(\theta_{1},\theta_{2}\right),\left(\theta_{1}>-90^{{}^{\circ}}\wedge \theta_{2}>-90^{{}^{\circ}}\right)\text{,}\\ \theta_{1}+360^{{}^{\circ}},\left(\theta_{1}<-90^{{}^{\circ}}\right)\text{,}\\ \theta_{2}+360^{{}^{\circ}},\left(\theta_{2}<-90^{{}^{\circ}}\right)\text{,} \end{array}\right) \end{array}$$

(11) $$\begin{array}{c} \theta_{2-value}=\left\{\begin{array}{c} \min\;\left(\theta_{1},\theta_{2}\right),\left(\theta_{1}>-90^{{}^{\circ}}\wedge \theta_{2}>-90^{{}^{\circ}}\right)\text{,}\\ \theta_{2},\left(\theta_{1}<-90^{{}^{\circ}}\right)\text{,}\\ \theta_{1},\left(\theta_{2}<-90^{{}^{\circ}}\right)\text{.} \end{array}\right) \end{array}$$

## 3. Patient-Specific Exercises

The rehabilitation process involves different rehabilitation exercises, for different patients going through different stages of recovery [35]. In this work, we developed the patient-passive exercise and patient-cooperative exercise, respectively. The patient-passive exercise is used at the early stage of therapy, during which the upper limb is driven along different predefined trajectories to help patients regain their limited range of motion (ROM); the patient-cooperative exercise is utilized at the later stage of recovery, during which a specially designed virtual game was designed to provide patients a more entertaining therapy experience, promoting them to put in their own efforts into the exercises.

### 3.1. Patient-Passive Exercise in Three DOFs

**Figure 4:**
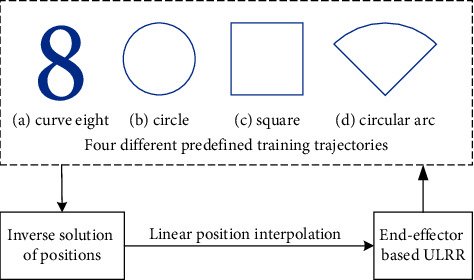
Process of patient-passive exercise with four different trajectories.

The entire implementation process of the developed patient-passive exercise is shown in Figure 4. Four different trajectories, curve eight, circle, square, and circular arc, are designed to guide patients to complete the patient-passive rehabilitation training in the horizontal plane, which can help them regain their lost motor functions of their upper limbs.

The designed trajectories are mapped to the motions of two horizontal servo motors according to the abovementioned inverse solution of positions, which are subsequently discretized into dozens of data points and interpolated by the HDT servo drives by using their linear position interpolation mode, during which the drive follows linear interpolation from point to point during the time period set, and for this type of interpolation, the master only has to send the position data. The third servo motor is used to adjust the vertical direction of the height, to adapt to the height of human rehabilitation training. It should be noted that the selection of rehabilitation training trajectories, the period of training, and the speed of rehabilitation training are manually adjusted by the rehabilitation physicians. In addition, the four designed trajectories are also used for evaluation test of the rehabilitation training effect.

### 3.2. Patient-Cooperative Exercise Based on Spring Model

The patient-cooperative exercise is an active rehabilitation training mode during which the patient drives the robot to complete the corresponding tasks according to the instructions of the HMI, such as playing games or drawing tracks; this can stimulate the enthusiasm of patients to participate in rehabilitation training. The inspiration of the patient-cooperative exercise in this work is from the admittance controller we conducted on ankle rehabilitation training [34], with its human-robot coupling system equivalent to a mass-spring-damp system as in (12), where *F* is the human-robot interaction force, *M* is the mass, *K* is the stiffness coefficient, and *B* represents the damping coefficient.

(12) $$\begin{array}{c} F\left(t\right)-Kx\left(t\right)-B\frac{dx\left(t\right)}{dt}=M\frac{dx^{2}\left(t\right)}{dt^{2}}\text{.} \end{array}$$

For the reason that the running speed of the end-effector type ULRR is slow and almost uniform during the rehabilitation process, its acceleration can be approximately zero, that is, d*x*^2^(*t*)/d*t*^2^=0. In addition, the response speed of the underdamped second-order system is a little slow with high computational complexity; especially using the proportional and time-shifting methods [35], we simplified the human-robot coupling system model in this patient-cooperative exercise into a single spring system, as in (13), which can efficiently improve the response speed of the control system.

(13) $$\begin{array}{c} F\left(t\right)=Kx\left(t\right)\text{.} \end{array}$$

**Figure 5:**
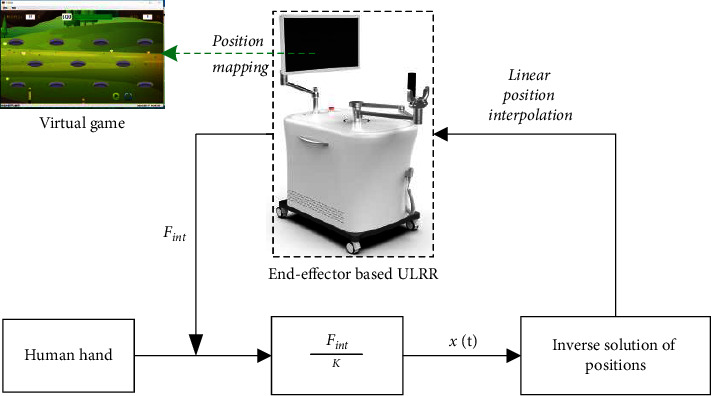
Process of the patient-cooperative exercise.

In this exercise mode, the end-effector type ULRR uses only three forces, *F*_*x*_, *F*_*y*_, and *F*_*z*_, which are measured from the six-axis circular load cell. The expected training trajectory of the terminal of the end-effector type ULRR can be obtained in real time by collecting the human-computer interaction force continuously, which can be tracked precisely based on the linear position interpolation. The entire implementation process of the developed patient-cooperative exercise is shown in Figure 5; the stiffness coefficient *K* is set to be 0.02 in the control system of the patient-cooperative exercise.

## 4. Experiments and Results

**Figure 6:**
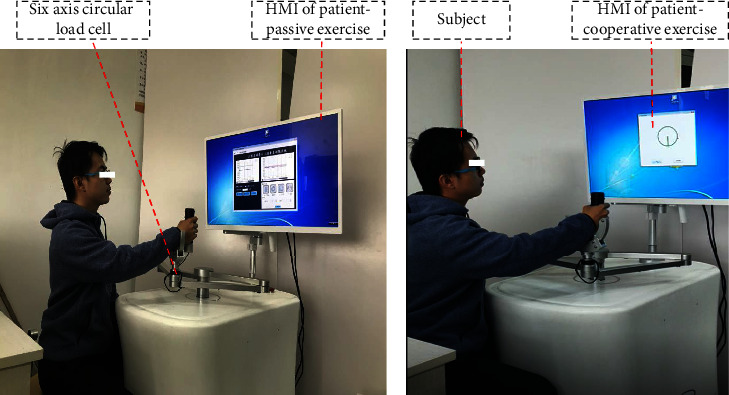
Experiments of patient-passive and patient-cooperative exercises. (a) Patient-passive exercise. (b) Patient-cooperative exercise.

To verify the effectiveness of the patient-specific exercises including the patient-passive exercise and patient-cooperative exercise, experiments have been conducted on our end-effector type ULRR with three healthy subjects (two males and one female, 23.33 ± 0.67 years old). All the subjects signed the informed consent form before the experiments. All experiments were approved by the Ethical Committee of Beijing University of Technology and conformed to the Declaration of Helsinki. The subjects sat next to the end-effector type ULRR, holding the handle (the grip force measurement sensor) during the experiments, as shown in Figure 6. The experiments of patient-passive exercise and patient-cooperative exercise were carried out, respectively. The sampling time of the interaction force from the six-axis circular load cell is 100Hz.

### 4.1. Trajectory Tracking of the Patient-Passive Exercise

As we mentioned above, there are four different trajectories designed to guide patients to complete the patient-passive exercise in the horizontal plane. To verify the trajectory tracking performance of our end-effector type ULRR's patient-passive exercise, we chose circular arc, square, and circle as the predefined trajectories to verify the performance, and two of the three subjects were selected to perform a form of training trajectory. The running speed of circle and circular arc is 50°/*s*, and the running speed of straight lines is 100mm/s.

**Figure 7:**
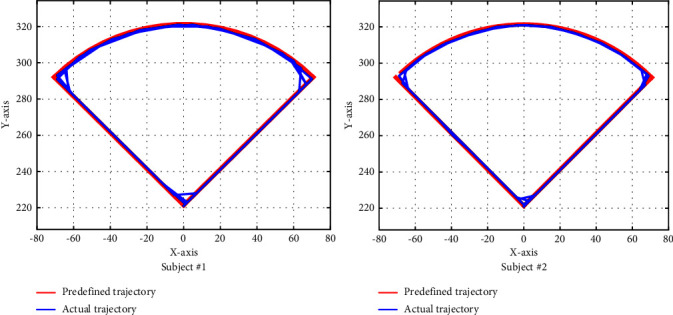
Performances of circular arc trajectory.

The starting point of the circular arc trajectory is (0,220); it goes counterclockwise in a straight line to point $\left(50\sqrt{2},220+50\sqrt{2}\right)$; after that, the trajectory is as in (14) in the counterclockwise direction to point $\left(-50\sqrt{2},220+50\sqrt{2}\right)$; then, it goes straight to point (0,220), circularly. The performance of the circular arc trajectory tracking is shown in Figure 7, where the red line represents the predefined trajectory while the blue line represents the actual trajectory.

(14) $$\begin{array}{c} \left\{\begin{array}{c} x=100\;\cos\;\theta \left(45^{{}^{\circ}}\leq \theta \leq 135^{{}^{\circ}}\right)\text{,}\\ y=100\;\sin\;\theta \left(45^{{}^{\circ}}\leq \theta \leq 135^{{}^{\circ}}\right)\text{.} \end{array}\right) \end{array}$$

**Figure 8:**
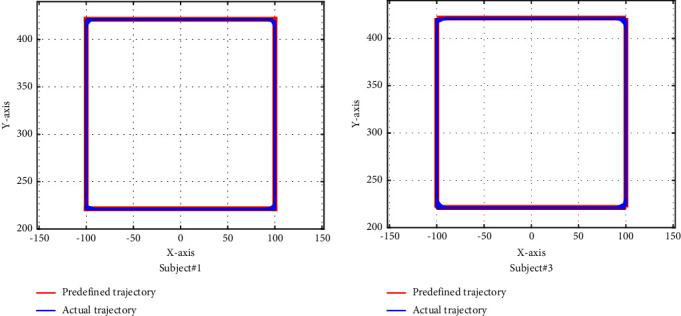
Performances of square trajectory.

The starting point of the square trajectory is also (0,220); then, it goes in a straight line in the counterclockwise direction to points (100,220), (100,420), (−100,420), (−100,220), and (0,220), circularly with the length of the side of 200mm. The trajectory tracking performance of the square trajectory is shown in Figure 8, where the red line and blue line represent the predefined and actual trajectories, respectively.

**Figure 9:**
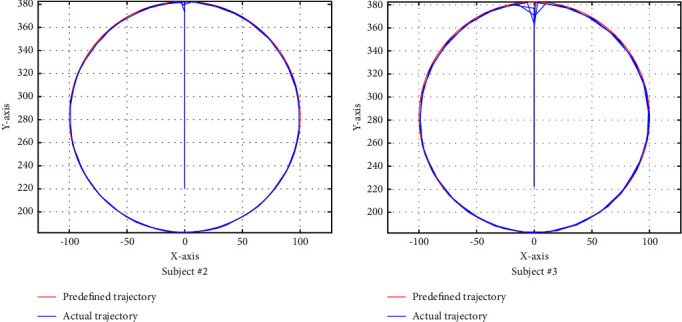
Performances of circle trajectory.

The starting point of the circle trajectory is also (0,220); then, it goes in a straight line to point (0,380); after that, the trajectory is as in (15) in the counterclockwise direction circularly. The trajectory tracking performance of the circle trajectory is shown in Figure 9, where the red line represents the predefined trajectory while the blue line represents the actual trajectory.

(15) $$\begin{array}{c} x^{2}+{\left(y-280\right)}^{2}=100^{2}\text{.} \end{array}$$

From Figures 7–9, we can see that the trajectory tracking accuracy of the three predefined trajectories at most moments is very high except at the corner where some tracks are inconsistent. In order to quantitatively describe the trajectory tracking error, we calculated the mean deviation (MD) and the root mean square deviation (RMSD) of the circle trajectory in the horizontal *x* direction from subject #2, where MD=∑_*i*=1_^*n*^|*x*_*p*_ − *x*_*a*_|/*n*, $RMSD=\sqrt{\sum_{i=1}^{n}{\left(x_{p}-x_{a}\right)}^{2}/n}$, with results showing that the MD is 0.8676 and the RMSD is 1.2643. For the reason that the trajectory deviation is very small and the actual trajectory is very close to the predefined trajectory, it will not cause harm to the limbs.

### 4.2. Interactive Performance of Patient-Cooperative Exercise

To verify the interactive and real-time performances of the developed patient-cooperative exercise, experiments have been conducted with the three healthy subjects through active circle testing by reference to the HMI. The starting point of the circle trajectory is (0,220); then, it goes in a straight line to point (0,320); after that, the reference trajectory is as in (16) in the counterclockwise direction circularly.

(16) $$\begin{array}{c} x^{2}+{\left(y-220\right)}^{2}=100^{2}\text{.} \end{array}$$

**Figure 10:**
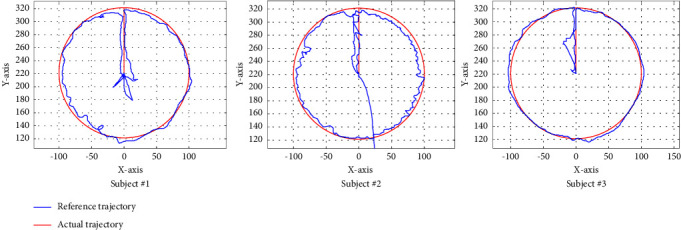
Performances of patient-cooperative exercise.

The three subjects drove the handle of the end-effector type ULRR to simulate the trajectory along the circle by referring to the circular trajectory on the HMI. Results of the patient-cooperative exercise of the three subjects are shown in Figure 10, where the red line represents the reference trajectory while the blue line represents the actual trajectory.

From the experimental results, we can see that although the actual trajectory did not completely follow the reference trajectory, the entire actual trajectory curve is smooth, and most of it runs along the reference trajectory, which verifies that the developed patient-cooperative exercise is very effective and flexible and has good real-time performance.

### 4.3. Discussion of the Experimental Results

To better demonstrate the advantage of our end-effector type ULRR and the developed patient-specific exercises, we compare our ULRR with one of the most latest and recognized PARRs known, the BULReD [2, 32]. At the mechanical level, like most upper limb rehabilitation robots, the BULReD only has two DOFs, while ours can achieve three DOFs by using a four-bar mechanism and a lifting mechanism. At the control system level, the BULReD used compliance control strategy based on measured human-robot interaction force and human users' position within subject-specific workspace to modify predefined training trajectories [32] and completed the bilateral coordination training [2], while we simplified the human-robot coupling system model into a single spring system instead of the mass-spring-damp system we conducted on ankle rehabilitation [34], and this helps reduce the computational complexity and efficiently improves the response speed of the control system. Although no quantitatively statistical analysis and comparative analysis have been carried out, our end-effector type ULRR is an improvement on the existing ULRRs in terms of mechanism design and the response speed of the control system from the perspective of experimental results.

## 5. Conclusions and Future Works

Robot-assisted rehabilitation training contributes significantly to the effectiveness of treatment, and they can alleviate manual therapy problems in terms of labor intensiveness, precision, and subjectivity [37]. As the end-effector type ULRR has the advantages of simple structures and less complex control algorithms and can avoid abnormal motion and posture of the target anatomical joints and specific muscles [34], we developed a three-DOF end-effector type ULRR with a one-DOF vertical movement and a parallelogram mechanism-based two-DOF horizontal movement. The mechanical design, control system, and inverse solution of positions of the developed ULRR were introduced, and the patient-specific exercises including patient-passive exercise with four different exercise trajectories and the patient-cooperative exercise based on the spring model were developed. Experimental results have shown that the developed patient-specific exercises with our new developed end-effector type ULRR have good trajectory tracking performance in patient-passive exercise and have flexible and good real-time performance in patient-cooperative exercise. The advantage of the mechanical design is that the designed end-effector type ULRR can achieve three DOFs by using a four-bar mechanism and a lifting mechanism, and the advantage of the patient-cooperative exercise is that we simplified the human-robot coupling system model into a single spring system instead of the mass-spring-damp system, which efficiently improves the response speed of the control system.

Future works will mainly start from the following aspects. One is to study the human-in-the-loop optimization control-based rehabilitation to perform more scientific rehabilitation training strategy [38]. The second is to study the intelligent prescription; the ULRR system should be able to adaptively and intelligently give the optimal training prescription based on the assessment of the patient's rehabilitation status. The third is to increase a large number of tests with healthy subjects to verify the safety, stability, and effectiveness of the entire system, to ensure that its performance and stability are good enough for upper limb disabled patients, so as to conduct clinical trials as soon as possible.

## Data Availability

The datasets analyzed in this article are not publicly available. Requests to access the datasets should be directed to the corresponding author.
